# Gene Editing for the Treatment of Hypercholesterolemia

**DOI:** 10.1007/s11883-024-01198-3

**Published:** 2024-03-18

**Authors:** Menno Hoekstra, Miranda Van Eck

**Affiliations:** 1 Division of Systems Pharmacology and Pharmacy, Leiden Academic Centre for Drug Research, Leiden University, Leiden, The Netherlands; 2 Pharmacy Leiden, Leiden, The Netherlands

**Keywords:** Dyslipidemia, Gene therapy, Liver, Gene editing, Hypercholesterolemia, Cardiovascular disease

## Abstract

**Purpose of Review:**

Here, we summarize the key findings from preclinical studies that tested the concept that editing of hepatic genes can lower plasma low-density lipoprotein (LDL)-cholesterol levels to subsequently reduce atherosclerotic cardiovascular disease risk.

**Recent Findings:**

Selective delivery of clustered regularly interspaced short palindromic repeats (CRISPR)/CRISPR-associated protein 9 (Cas9)-mediated gene editing tools targeting proprotein convertase subtilisin/kexin type 9 (PCSK9) to hepatocytes, i.e., through encapsulation into N-acetylgalactosamine-coupled lipid nanoparticles, is able to induce a stable ~ 90% decrease in plasma PCSK9 levels and a concomitant 60% reduction in LDL-cholesterol levels in mice and non-humane primates. Studies in mice have shown that this state-of-the-art technology can be extended to include additional targets related to dyslipidemia such as angiopoietin-like 3 and several apolipoproteins.

**Summary:**

The use of gene editors holds great promise to lower plasma LDL-cholesterol levels also in the human setting. However, gene editing safety has to be guaranteed before this approach can become a clinical success.

## Introduction

Atherosclerotic cardiovascular disease is characterized by the narrowing of the vessel lumen due to the accumulation of cholesterol and inflammatory cells within the arterial wall. The progression of this disease puts human subjects at risk of developing (clinical) conditions such as coronary heart diseases (myocardial infarction and angina), cerebrovascular diseases (transient ischemic attack and stroke), as well as life-threatening aortic pathologies (e.g. abdominal aortic and descending thoracic aneurysms). Having a relatively high plasma level of cholesterol transported by low-density lipoproteins (LDL), also commonly referred to as hypercholesterolemia, is an established risk factor for the development of atherosclerotic cardiovascular disease [1, 2]. Importantly, according to the *Heart Disease and Stroke Statistics 2021 Update* from the American Heart Association, ~ 30% of all people in the USA exhibit hypercholesterolemia, i.e., with LDL-cholesterol levels ≥ 130 mg/dl (≥ 3.4 mM) [3]. Similarly, in the year 2021, 25% of all Dutch people aged 30–70 years old could be qualified as being hypercholesterolemic [4]. There is thus a clear need to population-wide lower plasma cholesterol levels to battle the worldwide cardiovascular disease burden. At present, treatment with statins to inhibit cholesterol synthesis remains the first-line therapy for people displaying elevated plasma LDL-cholesterol levels. However, it should be acknowledged that statin treatment reduces the incidence of events and mortality related to cardiovascular disease by only 17–27% [5]. Moreover, statin treatment frequently comes with side effects, i.e., myopathy, that instigate therapy discontinuation [6]. Newer cholesterol-lowering therapies such as proprotein convertase subtilisin/kexin type 9 (PCSK9) antibody treatment are very expensive and/or require regular medical interventions with the help of a trained professional, limiting patient welfare. Gene editing is able to—in one go—induce a permanent change in a subject’s genetic makeup, thereby potentially also altering the susceptibility to disease.

**Fig. 1:**
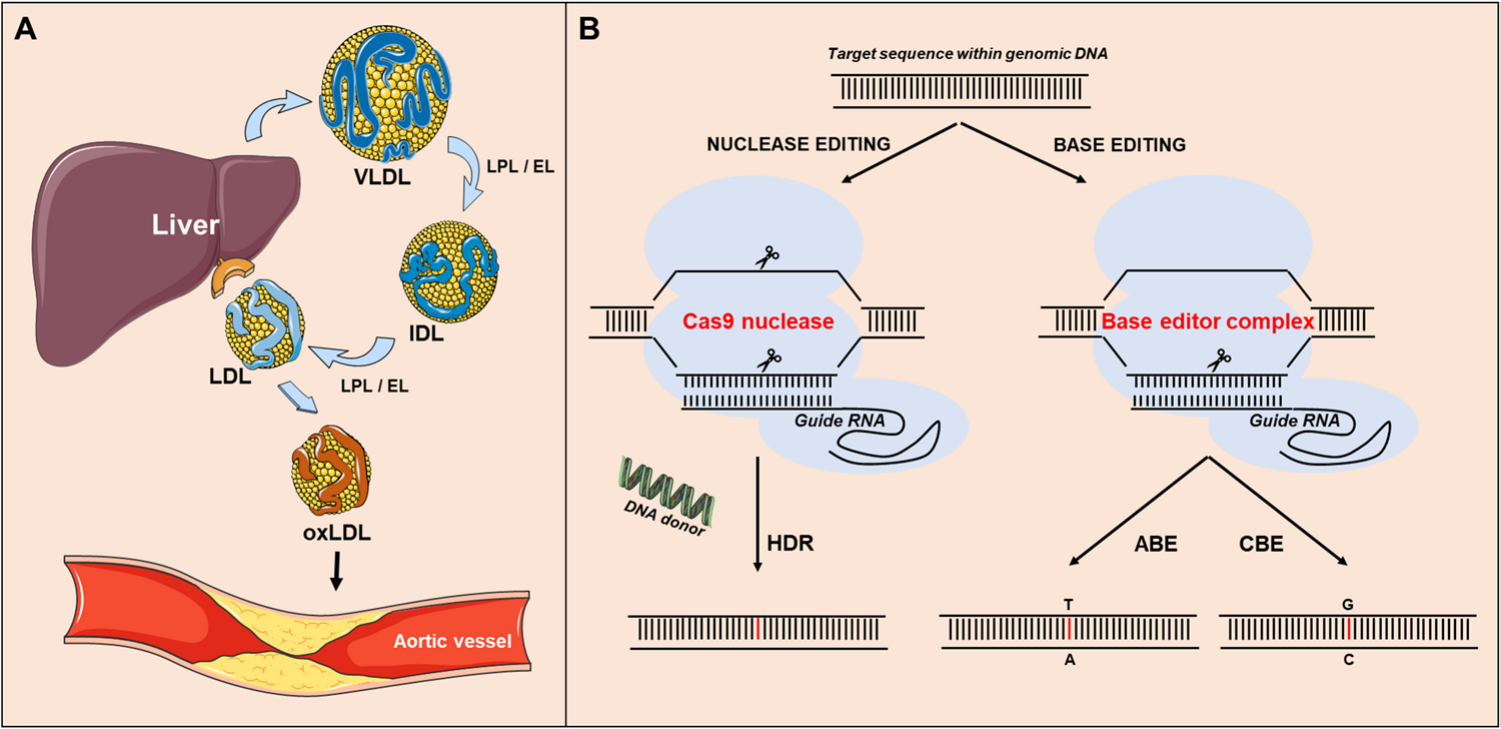
**A** Graphical representation of the relationship between low-density lipoprotein (LDL) metabolism and the development of atherosclerotic cardiovascular disease. The liver secretes cholesterol-containing very low-density lipoprotein (VLDL) particles that are rich in fatty acids packaged as triglycerides for delivery to metabolically active tissues such as the heart, muscles, and brown adipose tissue. Triglycerides are catabolized to glycerol and free fatty acids by the enzymes lipoprotein lipase (LPL) and endothelial lipase (EL) attached to the endothelium of the metabolic target tissues, which results in the subsequent conversion of the VLDL species into relatively smaller intermediate-density lipoprotein (IDL) and LDL particles, respectively. Under normolipidemic conditions, the cholesterol-rich LDL particles are rapidly cleared from the bloodstream via the LDL receptor present in the liver. However, when more LDL particles are present in the (hepatic) circulation than can be removed by the LDL receptors, relatively long-circulating LDL species become oxidized to generate pro-atherogenic oxLDL particles that can accumulate in atherosclerotic plaques and occlude the arterial lumen. **B** Schematic overview of CRISPR/Cas9-mediated approaches to change single nucleotides in the DNA. In the conventional nuclease editing approach, a Cas9 nuclease induces blunt-end double-strand breaks at the target site in the genomic DNA to enable the introduction of a nucleotide change by homology-directed repair (HDR). For this purpose, not only does a guide RNA need to bind with the protospacer adjacent motif sequence at the DNA target site but also the presence of an exogenous piece of DNA carrying the mutation is required to function as a repair template. In the newer base editing approach, a Cas9 nickase—a Cas9 protein variant with mutations in catalytic amino-acid residues in one of the two nuclease domains—with the help of a deaminase produces single-strand breaks in either the target or non-target DNA strand to subsequently facilitate, via hydrolytic deamination, an alanine (A) to guanine (G) conversion by an adenine base editor (ABE) or a cytosine (C) to thymidine (T) change with the use of a cytosine base editor (CBE). Image produced using freely available Servier medical art (https://smart.servier.com)

The liver is a key player in the modulation of plasma LDL-cholesterol levels as it (1) clears LDL particles from the blood circulation through receptor-mediated uptake by the LDL receptor present on hepatocytes and (2) secretes triglyceride-rich large very-low-density lipoproteins (VLDL) that can subsequently be converted into smaller cholesterol-rich LDL particles via lipoprotein lipase (LPL) and endothelial lipase (EL) mediated lipolysis (Fig. 1A). Impaired clearance of LDL particles by the liver can result in their oxidation and subsequent accumulation in atherosclerotic plaques within aortic vessels. As such, hepatic gene editing to beneficially impact LDL metabolism is a potentially interesting approach to overcome the development of hypercholesterolemia and associated atherosclerotic cardiovascular diseases. In this review, we will explain the rationale behind the technology currently utilized in liver-directed gene editing therapies, in general, and then highlight key findings from (landmark) preclinical studies in mice and non-human primates that have provided support for the application of gene editing for the treatment of high LDL-cholesterol levels in humans.

## Liver-Directed Gene Editing: From Adenoviral Gene Targeting Devices to Ligand-Coupled Lipid Nanoparticles

Hepatocytes, the primary cell type within the liver related to VLDL/LDL metabolism, are already for a long time of interest as a cellular target for gene-modifying therapeutic approaches in clinical disease. As summarized in the elegant review on the human liver-directed genome editing by Ginn et al. [7], already in the late 1990’s, clinical studies were executed with the aim to correct ornithine transcarbamylase deficiency—the most common genetic urea cycle disorder in humans—through adenovirus-mediated, hepatocyte-directed expression of a functional ornithine transcarbamylase gene variant. Although these initial trials showed severe off-target inflammatory effects and even lethality [8, 9], > 20 clinical studies have since been described using (advanced) adenoviral gene delivery techniques—i.e., with classical adenovirus types as well more advanced adeno-associated viruses (AAVs)—to evaluate the potential to cure patients from a variety of liver-associated genetic diseases such as for instance hemophilia B [10]. Notably, these adenovirus-based experimental approaches were largely dedicated to correct genetic deficiencies by adding a functional variant of a non-functioning gene to diseased cells. However, over the last decade, clustered regularly interspaced short palindromic repeats (CRISPR)/CRISPR-associated protein 9 (Cas9) gene editing strategies have been developed that are able to change individual nucleotides within a target DNA sequence. This has provided the opportunity to not only correct genetic deficiencies by improving gene functioning (gain-of-function modification) but also to make specific changes to the DNA that lead to a diminishment of the function of pathological proteins (loss-of-function modification). See Fig. 1B for a schematic overview of CRISPR/Cas9-mediated nuclease and base editing methods to induce or correct mutations in the DNA. Importantly, for this novel approach to be effective in permanently changing the function of genes, it is essential that both the Cas9 protein (derived from *Streptococcus pyogenes*) and the CRISPR guide RNA are present within the target cells, i.e., in hepatocytes. Given that the cargo capacity of AAV vectors (~ 4.7 kb total) is almost too limited to encapsulate both the Cas9 transcript of ~ 4 kb and the guide RNA sequence, researchers and pharmaceutical drug companies developing CRISPR/Cas9-based gene therapies have shifted their attention to higher capacity non-viral delivery devices such as lipid nanoparticles. Lipid nanoparticles are biodegradable carriers with an improved clinical safety profile as compared to AAV since they do not display the virus-associated immunogenicity. Furthermore, lipid nanoparticles cannot only transport Cas9 DNA, its guide RNAs, as well as potential accessory donor DNAs or proteins, but also be readily modified to deliver their cargo to specific tissues or individual cell types by attaching selected ligands. More specifically, supplying lipid nanoparticles with N-acetylgalactosamine (GalNAc), a high-affinity ligand for the asialoglycoprotein receptor located on hepatocytes, facilitates their selective delivery to the liver and improves the overall gene targeting efficiency [11–14]. Notably, the delivery refinement achieved through GalNAc coupling of the nanoparticles is not dependent on the presence of a functional LDL receptor [14]. As such, GalNAc-coupled lipid nanoparticles are currently regarded as state-of-the-art delivery vehicles for clinical use in CRISPR/Cas9-mediated gene editing therapies targeting hepatocytes.

## Studies in Mice Have Provided Proof-of-Concept for the Use of CRISPR/Cas9 Gene Editing Technology to Modulate Cholesterol Metabolism

The concept of treating hypercholesterolemia through hepatocyte-directed gene editing has been extensively tested in preclinical settings, with proof-of-concept studies primarily being executed in mice. Familial hypercholesterolemia is, with a global incidence of 1:200, a common inherited cause of high plasma LDL-cholesterol levels and early atherosclerotic cardiovascular disease development in humans [15]. Given that functional mutations within the LDL receptor represent the primary genetic cause of familial hypercholesterolemia, one would expect that much effort has been put into utilizing CRISPR/Cas9 technology to correct pathogenic defects in the LDL receptor prevalent in humans. However, to our surprise, we have been able to identify just one study in mice that aimed to prove that CRISPR-based gene editing is able to reverse hypercholesterolemia resulting from a functional defect in the LDL receptor, i.e., a E208X loss-of-function mutation [16]. In two other studies, mutations in the LDL receptor gene were introduced through AAV-mediated CRISPR guide RNA delivery that rather induced a deletion of normal protein functioning and led to the subsequent development of hypercholesterolemia in genetically normolipidemic wild-type or Cas9 transgenic mice [16, 17]. Human subjects carrying loss-of-function mutations in PCSK9 exhibit markedly lowered LDL-cholesterol levels and a striking 88% reduction in their cardiovascular disease risk as compared to non-affected individuals [18, 19]. Importantly, treatment of hypercholesterolemic patients with PCSK9 inhibitors such as evolocumab, alirocumab, and bococizumab has shown to be effective in reducing the risk for major adverse cardiovascular events [20]. Much effort has therefore been put on proving the potential of PCSK9 as a target in gene therapy for the treatment of hypercholesterolemia. Piloting studies carried out by Ding et al. showed that adenoviral delivery of a Cas9 protein and a PCSK9-targeting guide RNA to livers of C57BL/6 wild-type mice is able to achieve a substantial level of site-directed mutagenesis in the PCSK9 gene in vivo, resulting in a ~ 90% lowering of plasma PCSK9 protein levels [21]. The plasma PCSK9 lowering effect of PCSK9-targeting CRISPR guide RNAs could be effectively replicated by Ran et al., also using AAV-directed hepatic delivery of smaller Cas9 protein variants obtained from *Staphylococcus aureus* [22]. The studies by Ran et al. further validated the hypothesis that gene editing-mediated PCSK9 lowering can beneficially impact the hyperlipidemia extent. The authors observed that the ~ 95% decrease in plasma PCSK9 levels was paralleled by a ~ 40% decrease in total cholesterol levels [22]. Follow-up studies by Chadwick et al. showed that a significant reduction in plasma PCSK9 and cholesterol levels can also be obtained in C57BL/6 mice through adenoviral delivery of PCSK9-targeting base editors comprising CRISPR-Cas9 fused to a cytosine deaminase domain [23]. In light of the preference for nanoparticles over adenoviruses as delivery vehicles with respect to minimizing immunogenic and oncogenic gene therapy-associated side effects, Jiang et al. tested the potential to package the CRISPR / Cas9 components in lipid nanoparticles. They found that consecutive intravenous injections with a Cas9 mRNA-containing lipid nanoparticle and a single-guide RNA-containing lipid nanoparticle species resulted in a significant reduction in plasma PCSK9 protein levels in C57BL/6 mice without causing apparent damage to the liver [24]. The studies by Zhang et al. were, however, the first to show that it is feasible to package both the PCSK9-targeting single-guide RNA and Cas9 protein into one nanoparticle, which is considered a requisite for efficient translation of a gene editing approach to human clinical setting. More specifically, Zhang et al. combined the two gene-targeting components in an anionic complex encapsulated in a lipid shell covered with 4-aminophenyl β-d-galactopyranoside-modified polyethylene glycol phospholipids, the so-called Gal-LGCP nanoparticles. Treatment of mice with Gal-LGCP nanoparticles is associated with high-efficiency delivery of their cargo to hepatocytes, without parallel particle uptake by non-parenchymal liver cells (i.e., endothelial and Kupffer cells), and induces similar reductions in plasma PCSK9 protein and LDL-cholesterol levels as detected utilizing the previously mentioned virus-based and nanoparticle-aided gene delivery systems [25].

## CRISPR/Cas9-Mediated Editing of the PCSK9 Gene in the Liver Reduces LDL-Cholesterol Levels also in Non-human Primates

In light of the positive impact on plasma PCSK9 protein and LDL-cholesterol levels detected in mice, translational studies in non-human primates were readily initiated to bridge the gap towards clinical application of the PCSK9 gene editing technology. For this purpose, Wang et al. have generated AAV serotype 8 vectors carrying the PCSK9 gene targeting meganucleases M1 and M2 under the control of the liver-specific hyroxine-binding globulin promoter [26]. Intravenous administration of clinically relevant doses of the AAV8-M1PCSK9 to rhesus macaques induced dose-dependent and stable reductions in serum PCSK9 and LDL-cholesterol levels for over 200 days, with respective 84 and 60% reductions in high-dose-treated animals. Although Wang et al. provided the first proof for the concept that PCSK9 gene editing is able to beneficially impact plasma LDL-cholesterol levels also in non-human primates, the meganuclease-driven base editing technique, unfortunately, does not appear to be specific enough—as judged from the observed high-off-target editing rate with the M1 variant—and gives rise to an unwanted, chronic increase in blood transaminase levels [26]. The same research group has been able to improve the meganuclease-containing gene targeting vectors regarding their editing specificity through reducing the promoter length [27]. However, follow-up studies in macaques have suggested that liver-related toxicity will probably also remain an issue when AAVs containing the modified meganuclease promoter variants would be applied in human clinical settings [27]. Rothgangl. et al., therefore, switched to lipid nanoparticles as vehicle for transport of the PCSK9 gene-editing components to macaque livers. They observed that hepatic delivery of a chemically modified PCSK9-targeting single-guide RNA together with a 1-methoxyuridine-modified mRNA coding for an adenine base editor via high-dose lipid nanoparticle administration is able to achieve a PCSK9 gene editing efficiency of only 25% in adult cynomolgus macaques [28]. Also, relatively small reductions in plasma PCSK9 protein levels (− 39%) and LDL-cholesterol levels (− 19%) were thus measured after repeated administration of the base editor-containing lipid nanoparticles to adult cynomolgus macaques [28]. Rothgangl. et al. were also able to find antibodies directed against the introduced Cas9 protein in the blood of lipid nanoparticle-treated animals during the course of the study, indicative of an active immune response related to the treatment. T cell-mediated killing of Cas9-containing, successfully targeted hepatocytes may thus theoretically explain why the overall efficacy of their nanoparticle-based gene editing approach was rather limited. In support of the notion that lack of efficacy is not a common problem encountered with the use of lipid nanoparticles, a single intravenous infusion with lipid nanoparticles generated by Musunuru et al. that contained a newer generation base editor was able to induce high efficiency (> 60%) editing of the PCSK9 gene in livers of cynomolgus monkeys [29••]. As a result, Musunuru et al. showed that treatment of animals with a nanoparticle formulation containing both the adenine base editor 8.8-m mRNA and a PCSK9-targeting guide RNA is associated with a rapid and stable ~ 90% decrease in plasma PCSK9 levels and a concomitant 60% reduction in LDL-cholesterol levels [29••]. No data regarding the immune status of the monkeys after the PCSK9 gene editing treatment have been provided by Musunuru et al. However, since they only detected transient increases in indices of liver toxicity (shorter than 2 weeks; not related to the actual PCSK9 gene editing) in the context of a long-term (> 200 days) high editing efficiency [29••], it can be anticipated that the experimental approach of Musunuru et al. likely does not lead to pathological immune cell activation.

## Concluding Remarks and Future Outlook

From the preclinical findings discussed in this review, it can be anticipated that the use of nanoparticles carrying PCSK9-targeting gene editors holds great promise to be effective in lowering plasma PCSK9 protein and LDL-cholesterol levels in the human clinical setting. It is therefore not surprising that drug companies are in the process of evaluating their CRISPR/Cas9 gene editor-based PCSK9 lowering therapies in a relevant human population. For instance, VERVE Therapeutics is currently testing their lead therapy VERVE-101 in heterozygous familial hypercholesterolemia patients (clinical trial NCT05398029). In addition, CRISPR Therapeutics is in the process of preclinically optimizing/finalizing their PCSK9-targeting gene editing product CTX330 for future evaluation in clinical trials. A positive outcome of the human gene editing trials will not only provide the definite proof for the concept that gene editing can serve as treatment of high LDL-cholesterol levels but also open up the window for follow-up studies regarding the impact on human atherosclerotic cardiovascular disease risk.

Genome-wide association studies and validation studies in preclinical models have shown that genetic variation in many other hepatocyte-expressed genes may contribute to the development of dyslipidemia in humans [30]. Notably, VERVE Therapeutics and CRISPR Therapeutics currently also employ in a human setting the preclinically validated concept of nanoparticle-mediated CRISPR/Cas9-based lowering of the activity of the lipoprotein and endothelial lipase inhibitor angiopoietin-like 3 (ANGPTL3) to lower blood lipid levels [14, 31, 32]. Preclinical studies in rabbits and in hamsters have also shown a lipid-lowering and anti-atherogenic effect of genetically targeting hepatocyte-derived apolipoprotein C3 (APOC3) [33, 34]. Furthermore, Jarrett et al. have shown in mice that the gene coding for apolipoprotein B (APOB), the primary protein constituent of VLDL and LDL particles, can also be effectively edited using CRISPR/Cas technology to lower plasma cholesterol levels [17]. The state-of-the-art gene editing technology thus appears to have great potential to be further extended to include additional therapeutic targets. An overview of lipid metabolism-related gene targets currently already tested in CRISPR/Cas9-driven editing approaches is provided in Table 1.

**Table 1:** Overview of lipid metabolism-related gene targets utilized in CRISPR/Cas9 editing approaches

Gene target	Protein function	Gene editing status	References
ANGPTL3	Inhibitor of lipolysis	Preclinical: loss-of-function mutation induction achieved in mice and non-human primates Clinical: human gene editing trials are initiated by VERVE Therapeutics and CRISPR Therapeutics	[14, 31, 32]
APOB	Component of VLDL/LDL particles	Preclinical: loss-of-function mutation induction achieved in mice	[17]
APOC3	Inhibitor of lipolysis	Preclinical: loss-of-function mutation induction achieved in hamsters and rabbits	[33, 34]
LDLR	Hepatic uptake of LDL particles	Preclinical: both gain-of-function and loss-of-function mutation inductions achieved in mice	[16, 17]
PCSK9	Inhibitor of LDL receptor recycling	Preclinical: loss-of-function mutation induction achieved in mice and non-human primates Clinical: therapeutic efficacy of gene editing approach currently tested in heterozygous familial hypercholesterolemia patients (trial NCT05398029 by VERVE Therapeutics). CRISPR Therapeutics is also initializing a human trial	[21–28, 29••]

It should be acknowledged that the eventual benefit of the newly developed CRISPR/Cas9 gene therapies for patients at risk is not only dependent on scientific advancements, i.e., proper gene target selection and in vivo editing. The use of this novel technology should also have an acceptable cost/benefit ratio for society as a whole while remaining lucrative for further development by drug companies. To achieve this goal, it is of utmost importance that drug companies and healthcare professionals, in collaboration with scientists and the general public, provide answers to several questions. For instance, it has to be decided which specific populations should be targeted by the gene editing therapy. Will only human subjects at high risk due to genetic causes, i.e., familial hypercholesterolemia patients, get access to the lipid-lowering gene editing therapy or will drug companies and healthcare professionals be allowed by regulatory agencies (e.g. the Federal Drug Administration FDA or the European Medicine Agency EMA) to apply this novel therapeutic approach more broadly? Given that the costs for already approved gene therapies (i.e., for sickle cell anemia) generally exceed 1 million dollars per patient, it will also be important to verify that the gene editing can really eliminate the necessity for additional lipid-lowering therapies to effectively lower the risk of developing atherosclerotic cardiovascular disease. Furthermore, it remains uncertain as to whether the general public can be convinced that the application of CRISPR/Cas9 technology to permanently affect an individual’s genetic makeup is safe. Will people be able to trust that this new form of gene therapy does not induce off-target pathological gene edits or induce the inflammatory conditions, i.e., leukemia [35], seen in past adenoviral gene therapy-related trials? In this context, it is good to acknowledge that the original skepticism regarding the use of newly developed mRNA vaccines to protect people from SARS-CoV2 infection has been largely overturned by the eventual beneficial clinical outcome, i.e., the treatment-associated rapid decline of the global SARS-CoV2 pandemic. As such, it will be important to educate the general public on the long-term detrimental consequences of hyperlipidemia and promising lipid-lowering results and safety profiles seen in, for instance, the current clinical trial from VERVE Therapeutics to motivate patients to potentially make use of their gene editing approach in the (near) future.

## Data Availability

No datasets were generated or analyzed during the current study.
